# Supplementary material for: Association between mental illness and disciplinary confinement and its effect on mental health: A systematic review and meta-analysis
Source: PLoS One. 2025 Jun 10;20(6):e0325508. doi: 10.1371/journal.pone.0325508 (PMC12151385; doi:10.1371/journal.pone.0325508)
Supplement: S1 Text — Electronic search strategy for the systematic review and meta-analysis conducted. A search in Google Scholar and bibliographies enabled the identification of additional k = 92 studies. S2 Table. Details of the retrieved studies included. Association between mental disorders of inmates and placement into disciplinary confinement. S3 Table. Details of the retrieved studies included. Effects of disciplinary confinement on the mental health of inmates with or without pre-existing psychiatric conditions. S4 Table. PRISMA Checklist. From: Page MJ, McKenzie JE, Bossuyt PM, Boutron I, Hoffmann TC, Mulrow CD, et al. The PRISMA 2020 statement: an updated guideline for reporting systematic reviews. BMJ 2021;372:n71. https://doi.org/10.1136/bmj.n71. (DOCX) [file pone.0325508.s001.docx]

**Supplementary Online Content**

Giguère, Dellazizzo, Giguère & Dumais. Association between mental illness and disciplinary confinement and its effect on mental health: A systematic review and meta-analysis

**Table S1.** Electronic search strategy for the systematic review and meta-analysis conducted.

**Table S2-S3.** Details of the retrieved studies included.

**Table S4.** PRISMA Checklist.

| **Table S1.** Electronic search strategy for the systematic review and meta-analysis conducted through October 2023 | |
| --- | --- |
| Database; search | Search Terms |
| PubMed;  *k* = *2120* | ((("Solitary confinement"[Title/Abstract] OR segregation[Title/Abstract] OR seclusion[Title/Abstract] OR OR “disciplinary segregation”[Title/Abstract] OR “disciplinary confinement”[Title/Abstract] OR “punitive segregation”[Title/Abstract] OR "special housing unit"[Title/Abstract] OR "restrictive housing"[Title/Abstract] OR supermax[Title/Abstract] OR "maximum units"[Title/Abstract] OR "isolation units"[Title/Abstract]) AND (("mental health"[Title/Abstract] OR "psychological impact"[Title/Abstract] OR "psychological effect"[Title/Abstract] OR "behavioral impact"[Title/Abstract] OR "behavioral effect"[Title/Abstract] OR "medical impact"[Title/Abstract] OR "medical effect"[Title/Abstract] OR “psychological functioning”[Title/Abstract] OR “mental functioning”[Title/Abstract] OR “psychosocial functioning”[Title/Abstract] OR Depression[Title/Abstract] OR anxiety[Title/Abstract] OR loneliness[Title/Abstract] OR hallucinations[Title/Abstract] OR anger[Title/Abstract] OR aggressivity[Title/Abstract] OR irritability[Title/Abstract] OR violent[Title/Abstract] OR violence[Title/Abstract] OR nervousness[Title/Abstract] OR headaches[Title/Abstract] OR lethargy[Title/Abstract] OR tiredness[Title/Abstract] OR "Trouble sleeping"[Title/Abstract] OR insomnia[Title/Abstract] OR "heart palpitations"[Title/Abstract] OR "loss of appetite"[Title/Abstract] OR dizziness[Title/Abstract] OR nightmares[Title/Abstract] OR tremors[Title/Abstract] OR fainting[Title/Abstract] OR ruminations[Title/Abstract] OR "social withdrawal"[Title/Abstract] OR alienation[Title/Abstract] OR mood[Title/Abstract] OR suicide[Title/Abstract] OR mortality[Title/Abstract] OR Oversensitivity[Title/Abstract] OR hypersensivity[Title/Abstract]))) |
| PsycINFO;  *k = 2198* | (Abstract:((("solitary confinement") OR ("disciplinary confinement") OR ("disciplinary segregation") OR ("punitive segregation") OR (segregation) OR (seclusion) OR ("special housing unit") OR ("secure housing unit") OR ("restrictive housing") OR (supermax) OR ("maximum units") OR ("isolation units")) AND (("Physiological effect") OR ("Physiological impact") OR ("mental health") OR ("psychological impact") OR ("psychological functioning") OR ("psychosocial functioning") OR ("mental functioning") OR ("psychological effect") OR ("behavioral impact") OR ("behavioral effect") OR ("medical impact") OR ("medical effect") OR (anxiety) OR (depression) OR (loneliness) OR (hallucinations) OR (anger) OR (aggressivity) OR (irritability) OR (violent) OR (violence) OR (nervousness) OR (headaches) OR (lethargy) OR (tiredness) OR (“Trouble sleeping") OR (insomnia) OR ("heart palpitations") OR ("loss of appetite") OR (dizziness) OR (nightmares) OR (tremors) OR (fainting) OR (ruminations) OR ("social withdrawal") OR (alienation) OR (mood) OR (suicide) OR (mortality) OR (oversensitivty) OR (hypersensitivity)))) OR *same terms in Title.* |
| Web of Science;  *k = 5930* | (TS=(“Solitary confinement” OR segregation OR seclusion OR “disciplinary segregation” OR “disciplinary confinement” OR “punitive segregation” OR “special housing unit” OR “restrictive housing” OR supermax OR “maximum units” OR “isolation units” )) AND (TS=(“Physiological effect” OR “physiological impact” OR “mental health” OR “psychological functioning” OR “psychosocial functioning” OR “mental functioning” OR “psychological impact” OR “psychological effect” OR “behavioral impact” OR “behavioral effect” OR “medical impact” OR “medical effect” OR Depression OR anxiety OR loneliness OR hallucinations OR anger OR aggressivity OR irritability OR violent OR violence OR nervousness OR Headaches OR Lethargy OR tiredness OR “Trouble sleeping” OR insomnia OR “Heart palpitations” OR “Loss of appetite” OR Dizziness OR Nightmares OR tremors OR fainting OR ruminations OR “social withdrawal” OR alienation OR mood OR suicide OR mortality OR Oversensitivity OR hypersensivity)) |
| A search in **Google Scholar and bibliographies** enabled the identification of additional k= 92 studies. | |

Table S2. Details of the retrieved studies included. Association between mental disorders of inmates and placement into disciplinary confinement

| Author, date | Country | Study design | Correctional setting | Sample size  (% men) | Definition of psychiatric disorders or mental health services  (provided by included articles) | Methods to assess services and psychiatric disorders | Control group | Results (provided by articles) | Confounding factors | Quality of evidence | | | | | |
| --- | --- | --- | --- | --- | --- | --- | --- | --- | --- | --- | --- | --- | --- | --- | --- |
|  |  |  |  |  |  |  |  |  |  | Study limitations | Consistency | Directness | Precision | Publication bias | Quality of evidence |
| Butler,  2017 | USA | Cross-sectional | 242 state and federal | 6 074  (NR) | Mental health problems are defined as having been admitted to a mental hospital, taken medication for a mental illness, or received mental health counseling or other mental health services in the year immediately preceding their arrest | Survey | No mental health problems | b=0.05, SE=0.09 | None | Very serious limitations | No serious inconsistency | No serious indirectness | No serious imprecision | Undetected | Low  ⊕⊕⭘⭘ |
| Clark,  2018 | USA | Cross-sectional | State and Federal | 5 823  (83) | Been diagnosed by a medical professional prior to incarceration with various mental disorders: depressive, psychotic, personality, manic/bipolar, posttraumatic stress disorder (PTSD), anxiety, or any other disorder | Survey | No mental illness | OR=1.36, SE=0.07 | Type of misconduct, age, ethnicity, sex, marital status, learning disability, education, prior employment, prior, homeless, prior physical abuse, prior sexual abuse, substance use, prior arrests, prior incarcerations, instant offense | Serious limitations | No serious inconsistency | No serious indirectness | No serious imprecision | Undetected | Moderate  ⊕⊕⊕⭘ |
| Coid,  2003 | England and Wales | Cross-sectional | 131 prisons | 3 141  (75) | Admission psychiatric hospital | Survey | No previous experiences of psychiatric  hospitalization or treatment prior to imprisonment | Men  OR=0.89, CI=0.61;1.30  Women  OR=1.21, CI=0.73;2.01 | Age, prisoner type, marital status, social class, strip conditions, previous prison, time in prison ≥1 year | Serious limitations | No serious inconsistency | No serious indirectness | No serious imprecision | Undetected | Moderate  ⊕⊕⊕⭘ |
|  |  |  |  |  | Psychiatric hospital  < 6-months |  |  | Men  OR=1.55, CI=0.61;3.96  Women  OR=0.86, CI=0.25;2.94 |  |  |  |  |  |  |  |
|  |  |  |  |  | Court-ordered psychiatric treatment in prison |  |  | Men  OR=1.08, CI=0.77;1.52  Women  OR=1.23, CI=0.62;2.42 |  |  |  |  |  |  |  |
|  |  |  |  |  | Psychiatric treatment in prison |  |  | Men  OR=1.21, CI=0.91;1.61  Women  OR=1.86, CI=1.23;2.82 |  |  |  |  |  |  |  |
|  |  |  |  |  | Personality disorders Avoidant | SCID-II questionnaire for DSM IV personality disorders | No personality disorders | Men  OR=0.97, CI=0.69;1.37  Women  OR=1.06, CI=0.66;1.70 |  |  | No serious inconsistency |  | No serious imprecision |  |  |
|  |  |  |  |  | Dependant |  |  | Men  OR=0.89, CI=0.58;1.37  Women  OR=1.17, CI=0.60;2.27 |  |  |  |  |  |  |  |
|  |  |  |  |  | Obsessive-compulsive |  |  | Men  OR=0.91, CI=0.65;1.27  Women  OR=1.12, CI=0.67;1.87 |  |  |  |  |  |  |  |
|  |  |  |  |  | Paranoid |  |  | Men  OR=1.37, CI=1.10;1.71  Women  OR=1.58, CI=1.07;2.33 |  |  |  |  |  |  |  |
|  |  |  |  |  | Schizotypal |  |  | Men  OR=1.09, CI=0.82;1.45  Women  OR=0.99, CI=0.63;1.58 |  |  |  |  |  |  |  |
|  |  |  |  |  | Schizoid |  |  | Men  OR=0.93, CI=0.73;1.18  Women  OR=1.16, CI=0.78;1.73 |  |  |  |  |  |  |  |
|  |  |  |  |  | Histrionic |  |  | Men  OR=1.62, CI=0.76–3.47  Women  OR=0.50, CI=0.13;1.92 |  |  |  |  |  |  |  |
|  |  |  |  |  | Narcissistic |  |  | Men  OR=2.13, CI=1.25;3.61  Women  OR=0.71, CI=0.24;2.11 |  |  |  |  |  |  |  |
|  |  |  |  |  | Borderline |  |  | Men  OR=1.24, CI=0.93;1.64)  Women  OR=1.67, CI=1.07;2.61 |  |  |  |  |  |  |  |
|  |  |  |  |  | Anti-social |  |  | Men  OR=2.41, CI=1.87;3.10  Women  OR=2.34, CI=1.54;3.58 |  |  |  |  |  |  |  |
|  |  |  |  |  | Schizophrenia | Schedules for Clinical Assessment in Neuropsychiatry (SCAN) | No major mental disorders  using SCAN data | Men  OR=0.37, CI=0.03;4.01  Women (Unadjusted)  OR=3.21, CI=0.99;10.40 |  |  | Serious inconsistency |  | No serious imprecision |  | Low  ⊕⊕⭘⭘ |
|  |  |  |  |  | Depression  496  (78.3) |  |  | Men  OR=0.96, CI=0.43;2.17  Women  OR=2.39, CI=0.48;11.9 |  |  |  |  |  |  |  |
|  |  |  |  |  | Brain dysfunction |  |  | Men  OR=0.35, CI=0.04;3.23  Women (Unadjusted)  OR=1.80, CI=0.16;20.79 |  |  |  |  |  |  |  |
|  |  |  |  |  | Anxiety |  |  | Men  OR=1.77, CI=0.80;3.90  Women  OR=2.22, CI=0.50;9.73 |  |  | No serious inconsistency |  | Serious imprecision |  |  |
|  |  |  |  |  | Obsessive-compulsive |  |  | Men  OR=1.47, CI=0.25;8.60  Woman  NA |  |  |  |  |  |  |  |
| Henry, 2022 | USA | Cross sectional | State and federal | 5 228  (NR) | Learning disability | Survey | No disorders of mental health | OR=1.19, CI=0.91;1.56 | Type of rule violation for which the disciplinary action was received | Serious limitations | No serious inconsistency | No serious indirectness | No serious imprecision | Undetected | Moderate  ⊕⊕⊕⭘ |
|  |  |  |  |  | Any personality disorders |  |  | OR=0.76, CI=0.32;1.77 |  |  |  |  |  |  |  |
|  |  |  |  |  | Psychotic or bipolar disorder |  |  | OR=1.36, CI=0.88;2.09 |  |  |  |  |  |  |  |
|  |  |  |  |  | Other single other disorder (attention deficit disorder, attention deficit/hyperactivity disorder, anxiety disorder, depressive disorder, posttraumatic stress disorder, or other disorder) |  |  | OR=1.00, CI=0.79;1.26 |  |  |  |  |  |  |  |
|  |  |  |  |  | Multiple mental disorders |  |  | OR=1.22, CI=1.05;1.41 |  |  |  |  |  |  |  |
| Severson,  2019 | USA | Cross sectional | State and federal | 7 189  (82) | Lifetime histories of mental health problems were defined as ever having stayed overnight in a mental hospital, ever having used prescription medication, ever receiving mental health services from a qualified mental health professional, or ever receiving other mental health treatment or services | Survey | No lifetime histories of mental health problems | Men  OR=1.16. SE=0.08  Women  OR=1.08, SE=0.15 | Age, gender, ethnicity, education, prior employment, marital status, prior incarceration, current offense severity, time served, prison work assignment, visitation, alcohol and drug dependence, child or adult physical or sexual abuse | Serious limitations | No serious inconsistency | No serious indirectness | No serious imprecision | Undetected | Moderate  ⊕⊕⊕⭘ |

Table S3. Details of the retrieved studies included. Effects of disciplinary confinement on the mental health of inmates with or without pre-existing psychiatric conditions

| Author, date | Country | Study design | Correctional setting | Sample size  (% men) | Methods to assessed services and psychiatric disorders | Control group | Psychiatric symptoms  (provided by articles) | Results (provided by articles) | Confounding factors | Quality of evidence | | | | | |
| --- | --- | --- | --- | --- | --- | --- | --- | --- | --- | --- | --- | --- | --- | --- | --- |
|  |  |  |  |  |  |  |  |  |  | Study limitations | Consistency | Directness | Precision | Publication bias | Quality of evidence |
| Kaba,  2014 | USA | Retrospective, Longitudinal | Local detention facilities (New York Jail system) | 134 188  (90.8) | Department of Correction database | Not in solitary confinement | Self-harm defined as an act performed by individuals on themselves with the potential to result in physical injury | Without serious mental illness  OR = 10.15, CI=8.53; 12.08  With serious mental illness  OR=4.03, CI=3.10; 5.24 | None | Serious limitations | No serious inconsistency | No serious indirectness | No serious imprecision | ? | Moderate  ⊕⊕⊕⭘ |
|  |  |  |  |  |  |  | Potentially fatal self-harm defined as an act with a high probability of causing significant disability or death, regardless of whether death actually occurred | Without serious mental illness  OR= 6.16, CI=3.47; 10.96  With serious mental illness  OR=9.06, CI=4.03; 20.40 |  |  |  |  |  | ? |  |
| Miller,  1994  1997 | USA | Cross-sectional | Medium security federal correctional | 30  (100) | Brief Symptom Inventory | General correctional population, administrative segregation | Psychological distress | F=3.61, p<0.041 | None | Very serious limitations | ? | No serious indirectness | ? | ? | Very low  ⊕⭘⭘⭘ |
|  |  |  |  |  |  |  | obsessive-compulsive symptoms | F=5.80, p=0.01 |  |  |  |  |  |  |  |
|  |  |  |  |  |  |  | interpersonal sensitivity | F=4.84, p=0.02 |  |  |  |  |  |  |  |
|  |  |  |  |  |  |  | hostility | F=5.0, p=0.01 |  |  |  |  |  |  |  |
|  |  |  |  |  |  |  | depressive symptoms | F=1.04, p=0.37 |  |  |  |  |  |  |  |
|  |  |  |  |  |  |  | anxiety | F=1.59, p=0.22 |  |  |  |  |  |  |  |
|  |  |  |  |  |  |  | paranoid ideation | F=0.77, p=0.47 |  |  |  |  |  |  |  |
| Wildeman,  2020 | Denmark | Retrospective, Longitudinal | Closed prisons, open prisons, and jails | 36 360  (NR) | Administrative data | had received another type of disciplinary sanction, no recorded disciplinary actions | Contact with the mental health care system was defined as whether they were referred by their general physician to the mental health care system and used the referral | p=0.05 | None | Serious limitations | ? | No serious indirectness | ? | ? | Moderate  ⊕⊕⊕⭘ |
| Wynn, 2008 | USA | Cross-sectional  (these) | Supermax prisons and special housing units | 175  (NR) | By prison mental health staff upon admission or during incarceration | No previous or current mental health problem | Psychological distress (problems thinking, concentration or paying attention, thought disorders perceptual visual or auditory, self-mutilation, threatened suicide, attempted suicide, been sent to an observation cell or mental health unit, take one hour of recreation) | t(125) =-7.842, p=0.000 | None | Very serious limitations | ? | No serious indirectness | ? | ? | Very low  ⊕⭘⭘⭘ |
|  |  |  |  | 55  (NR) | Survey | No control group | Transferred to a psychiatric unit due to psychiatric deterioration, suicide threats or suicide attempts | 40% |  |  |  |  |  |  |  |
| Cloud, 2023 | USA | Cross-sectional | State  (Louisiana) | 517  (100) | Survey | Closed-cell confinement, protective custody, or death row | Self-injurious behavior | OR=1.97, CI=1.15; 3.53 | Nominal prospect of being released from prison, ethnicity, time in solitary confinement | Very serious limitations | No serious inconsistency | No serious indirectness | No serious imprecision | ? | Low  ⊕⊕⭘⭘ |

**Table S4.** PRISMA Checklist.

| **Section and Topic** | **Item #** | **Checklist item** | **Location where item is reported** |
| --- | --- | --- | --- |
| **TITLE** | | |  |
| Title | 1 | Identify the report as a systematic review. | 1 |
| **ABSTRACT** | | |  |
| Abstract | 2 | See the PRISMA 2020 for Abstracts checklist. | 2 |
| **INTRODUCTION** | | |  |
| Rationale | 3 | Describe the rationale for the review in the context of existing knowledge. | 3-4 |
| Objectives | 4 | Provide an explicit statement of the objective(s) or question(s) the review addresses. | 4 |
| **METHODS** | | |  |
| Eligibility criteria | 5 | Specify the inclusion and exclusion criteria for the review and how studies were grouped for the syntheses. | 5 |
| Information sources | 6 | Specify all databases, registers, websites, organisations, reference lists and other sources searched or consulted to identify studies. Specify the date when each source was last searched or consulted. | 5 |
| Search strategy | 7 | Present the full search strategies for all databases, registers and websites, including any filters and limits used. | Table S1 |
| Selection process | 8 | Specify the methods used to decide whether a study met the inclusion criteria of the review, including how many reviewers screened each record and each report retrieved, whether they worked independently, and if applicable, details of automation tools used in the process. | 5 |
| Data collection process | 9 | Specify the methods used to collect data from reports, including how many reviewers collected data from each report, whether they worked independently, any processes for obtaining or confirming data from study investigators, and if applicable, details of automation tools used in the process. | 5 |
| Data items | 10a | List and define all outcomes for which data were sought. Specify whether all results that were compatible with each outcome domain in each study were sought (e.g. for all measures, time points, analyses), and if not, the methods used to decide which results to collect. | 5 |
|  | 10b | List and define all other variables for which data were sought (e.g. participant and intervention characteristics, funding sources). Describe any assumptions made about any missing or unclear information. | 5 |
| Study risk of bias assessment | 11 | Specify the methods used to assess risk of bias in the included studies, including details of the tool(s) used, how many reviewers assessed each study and whether they worked independently, and if applicable, details of automation tools used in the process. | 6 |
| Effect measures | 12 | Specify for each outcome the effect measure(s) (e.g. risk ratio, mean difference) used in the synthesis or presentation of results. | 6 |
| Synthesis methods | 13a | Describe the processes used to decide which studies were eligible for each synthesis (e.g. tabulating the study intervention characteristics and comparing against the planned groups for each synthesis (item #5)). | 6 |
|  | 13b | Describe any methods required to prepare the data for presentation or synthesis, such as handling of missing summary statistics, or data conversions. | 6 |
|  | 13c | Describe any methods used to tabulate or visually display results of individual studies and syntheses. | 6 |
|  | 13d | Describe any methods used to synthesize results and provide a rationale for the choice(s). If meta-analysis was performed, describe the model(s), method(s) to identify the presence and extent of statistical heterogeneity, and software package(s) used. | 6 |
|  | 13e | Describe any methods used to explore possible causes of heterogeneity among study results (e.g. subgroup analysis, meta-regression). | 6 |
|  | 13f | Describe any sensitivity analyses conducted to assess robustness of the synthesized results. | 6 |
| Reporting bias assessment | 14 | Describe any methods used to assess risk of bias due to missing results in a synthesis (arising from reporting biases). | 6 |
| Certainty assessment | 15 | Describe any methods used to assess certainty (or confidence) in the body of evidence for an outcome. | 6 |
| **RESULTS** | | |  |
| Study selection | 16a | Describe the results of the search and selection process, from the number of records identified in the search to the number of studies included in the review, ideally using a flow diagram. | 7-10 |
|  | 16b | Cite studies that might appear to meet the inclusion criteria, but which were excluded, and explain why they were excluded. | 7-10 |
| Study characteristics | 17 | Cite each included study and present its characteristics. | 7-10, Table S2 |
| Risk of bias in studies | 18 | Present assessments of risk of bias for each included study. | 7-10 |
| Results of individual studies | 19 | For all outcomes, present, for each study: (a) summary statistics for each group (where appropriate) and (b) an effect estimate and its precision (e.g. confidence/credible interval), ideally using structured tables or plots. | 7-10 |
| Results of syntheses | 20a | For each synthesis, briefly summarise the characteristics and risk of bias among contributing studies. | 7-10 |
|  | 20b | Present results of all statistical syntheses conducted. If meta-analysis was done, present for each the summary estimate and its precision (e.g. confidence/credible interval) and measures of statistical heterogeneity. If comparing groups, describe the direction of the effect. | 7-10 |
|  | 20c | Present results of all investigations of possible causes of heterogeneity among study results. | 7-10 |
|  | 20d | Present results of all sensitivity analyses conducted to assess the robustness of the synthesized results. | 7-10 |
| Reporting biases | 21 | Present assessments of risk of bias due to missing results (arising from reporting biases) for each synthesis assessed. | 7-10 |
| Certainty of evidence | 22 | Present assessments of certainty (or confidence) in the body of evidence for each outcome assessed. | 7-10 |
| **DISCUSSION** | | |  |
| Discussion | 23a | Provide a general interpretation of the results in the context of other evidence. | 11-12 |
|  | 23b | Discuss any limitations of the evidence included in the review. | 11-12 |
|  | 23c | Discuss any limitations of the review processes used. | 12 |
|  | 23d | Discuss implications of the results for practice, policy, and future research. | 12 |
| **OTHER INFORMATION** | | |  |
| Registration and protocol | 24a | Provide registration information for the review, including register name and registration number, or state that the review was not registered. | NA |
|  | 24b | Indicate where the review protocol can be accessed, or state that a protocol was not prepared. | NA |
|  | 24c | Describe and explain any amendments to information provided at registration or in the protocol. | NA |
| Support | 25 | Describe sources of financial or non-financial support for the review, and the role of the funders or sponsors in the review. | 14 |
| Competing interests | 26 | Declare any competing interests of review authors. | 14 |
| Availability of data, code and other materials | 27 | Report which of the following are publicly available and where they can be found: template data collection forms; data extracted from included studies; data used for all analyses; analytic code; any other materials used in the review. | N/A |

*From:* Page MJ, McKenzie JE, Bossuyt PM, Boutron I, Hoffmann TC, Mulrow CD, et al. The PRISMA 2020 statement: an updated guideline for reporting systematic reviews. BMJ 2021;372:n71. doi: 10.1136/bmj.n71
