# Supplementary material for: Association between mental illness and disciplinary confinement and its effect on mental health: A systematic review and meta-analysis
Source: PLoS One. 2025 Jun 10;20(6):e0325508. doi: 10.1371/journal.pone.0325508 (PMC12151385; doi:10.1371/journal.pone.0325508)
Supplement: Attachment — Submitted filename: Response to Reviewers.docx [file pone.0325508.s002.docx]

**Reviewer #1:**

The author is suggested to do revisions as mentioned below:

1) Include only best keywords that describe your paper for indexing and web searches. The more discriminating your keywords are the greater is the likelihood that your article will be found and cited. For example replace “sys-tematic review”

**Authors’ response:** Thank you for the suggestion. We have replaced the term ‘systematic review’ by the keyword ‘inmates’.

2) The abstract is informative for the purpose, but it do not indicate the significant level of the focus highlighted. Provide more specific reason on how this study will offer a potential route, apart from other works in this area.

**Authors’ response:** Thank you for the suggestion. We added at the start and the end of the abstract more specific reasons on how this study will offer a potential route.

3) Personal biases were found at various sections in the paper. The author is suggested to modify the use of "We" in the article. Reform such sentences.

**Authors’ response:** Thank you for your suggestion, the sentences have been reworded.

4) Citations are found in the conclusion. References should be generally avoided in conclusions. If you have already cited the ideas earlier in the paper that you are summarizing in your conclusion, you do not need to cite them again.

**Authors’ response:** Thank you for your suggestion, sentences containing citations have been moved in the discussion.

5) The conclusion derived is not presented well. Modify the conclusion logically.

**Authors’ response:** Thank you for your comment, the conclusion has been revised.

6) Reformat the acknowledgment section. If Alexandre Dumais (AD) had any other role like funding mention it also.

**Authors’ response:** After rereading the content guidelines provided by the journal for the acknowledgments section, this section has been removed since source funding should not be mentioned there.

7) I suggest the author to add some new references by referring more works in the same field, especially the latest works of the year 2024 and indicate some.

**Authors’ response:** Thank you for your suggestion, the 5 following articles have been added:

- The results of the article by Wooldrege et al., 2024 have been added to the discussion for comparison with the present study (see lines 309-314).
- The results of the article by Vanhaesebrouck et al., 2024 have been added to the discussion (see lines 355-363).
- The studies by Karplus, 2023, Meyers et al., 2023 and Cloud et al., 2023 have been added to the discussion of reforms and alternative programs to limit the use of DC (see lines 366-373).

8) The motivation and significance on this paper needs to be further emphasized.

**Authors’ response:** Thank you for the suggestion. In addition to the modifications conducted following comment 2, we have added a paragraph at the end of the discussion to emphasize the significance of this paper for research and policy (see lines 408-421)

**Reviewer #2:**

The work is good but may be improved by the inclusion of the following suggestions.

1. The “Statistical analysis” section should be added to the manuscript.

**Authors’ response:** Please see the Statistical analysis section in the method (lines 126-151).

2. In the figures ‎the error bar or the standard deviation should be included.‎

**Authors’ response:** In Figure 2, the bar represents the confidence intervals associated with the odds ratio, which are also indicated in parentheses. We have added mention of the OR and the CI to the figure for clarification.

3. Many grammar errors could be found in the paper, please check and deeply revise them. ‎

**Authors’ response:** Thank you, the paper has been completely revised.

4. Please improve the novelty of statement with focus on the novelty.

**Authors’ response:** Thank you for your suggestion. We have added a sentence at the end of the introduction stating that this is the first systematic review and meta-analysis specifically on DC covering these topics (see lines 104-105). In addition, we have added sentences describing the novelty of the paper in the last paragraph of the discussion (see lines 411-414).

5. Quantitative information should be provided in the abstract.

**Authors’ response:** Information on the method and quantitative results can be found on lines 10-12 and 15-21 of the abstract.

6. Conclusion section should be rewritten to make it more quantitative.

**Authors’ response:** Thank you for your comments. We have added a summary of the effect sizes obtained.

7. All abbreviations were defined when used for the first time in the manuscript.

**Authors’ response:** Thank you for this observation, the abbreviations have been defined.

8. What about Strengths and limitations in your work? Please add it in discussion.

**Authors’ response:** We have reworded the paragraph of the discussion to better define the limitations of our research. We added a last paragraph in the discussion to also highlight the strengths of the manuscript.

9. Add your recommendations for future research.

**Authors’ response:** Thank you for your suggestion, several recommendations for future research have been added to the two last paragraphs of the discussion.

10. The last paragraph ‎of Introduction should include the study objectives/procedures in brief. ‎

**Authors’ response:** Thanks for your comment, we have reworded the objectives at the end of the introduction section to make them more clearly identifiable.
